# Neurofibromin Deficiency Causes Epidermal Growth Factor Receptor Upregulation through the Activation of Ras/ERK/SP1 Signaling Pathway in Neurofibromatosis Type 1-Associated Malignant Peripheral Nerve Sheet Tumor

**DOI:** 10.3390/ijms222413308

**Published:** 2021-12-10

**Authors:** Gun-Hoo Park, Su-Jin Lee, Chang-Gun Lee, Jeonghyun Kim, Eunkuk Park, Seon-Yong Jeong

**Affiliations:** 1 Department of Medical Genetics, Ajou University School of Medicine, Suwon 16499, Korea; gunhoopark5699@aumc.ac.kr (G.-H.P.); sujinlee5699@gmail.com (S.-J.L.); dangsunsang@ajou.ac.kr (C.-G.L.); kjh2828@ajou.ac.kr (J.K.); 2 Department of Biomedical Sciences, Ajou University Graduate School of Medicine, Suwon 16499, Korea

**Keywords:** neurofibromatosis type 1 (NF1), neurofibromin, EGFR, malignant peripheral nerve sheet tumor (MPNST), sarcoma, tumor progression

## Abstract

Neurofibromatosis type 1 (NF1) is an autosomal dominant human genetic disorder. The progression of benign plexiform neurofibromas to malignant peripheral nerve sheet tumors (MPNSTs) is a major cause of mortality in patients with NF1. Although elevated epidermal growth factor receptor (EGFR) expression plays a crucial role in the pathogenesis of MPNST, the cause of EGFR overexpression remains unclear. Here, we assessed EGFR expression levels in MPNST tissues of NF1 patients and NF1 patient-derived MPNST cells. We found that the expression of EGFR was upregulated in MPNST tissues and MPNST cells, while the expression of neurofibromin was significantly decreased. Manipulation of *NF1* expression by *NF1* siRNA treatment or NF1-GAP-related domain overexpression demonstrated that EGFR expression levels were closely and inversely correlated with neurofibromin levels. Notably, knockdown of the *NF1* gene by siRNA treatment augmented the nuclear localization of phosphorylated SP1 (pSP1) and enhanced pSP1 binding to the *EGFR* gene promoter region. Our results suggest that neurofibromin deficiency in NF1-associated MPNSTs enhances the Ras/ERK/SP1 signaling pathway, which in turn may lead to the upregulation of EGFR expression. This study provides insight into the progression of benign tumors and novel therapeutic approaches for treatment of NF1-associated MPNSTs.

## 1. Introduction

Neurofibromatosis type 1 (NF1), a genetic disease, is caused by a novel germline mutation on the *NF1* gene or hereditary transmission of *NF1* mutations [1]. The *NF1* tumor suppressor gene encodes a neurofibromin that negatively regulates of Ras by switching Ras-GTP to Ras-GDP [2]. Loss of neurofibromin promotes activation of the Ras effector pathways and phosphoinositide 3-kinase (PI3K) pathways, which are engaged in the control of cell proliferation, survival, and metabolism [3]. In addition, neurofibromin involves the cAMP pathway via G protein-coupled receptor (GPCR)-mediated adenylyl cyclase activation [4]. Patients with NF1 have a variety of symptoms that affect the skin, bone, peripheral nervous system, and soft tissues [5]. The clinical features of NF1 are variable and unpredictable, even in the same family [6]. The clinical hallmark of NF1 is the neurofibroma, a slow-growing benign tumor [7]. This tumor progression is related with loss of heterozygosity of *NF1* gene function in the Schwann lineage cells [8]. Half of all patients with NF1 are affected with benign plexiform neurofibromas (PNs) [9]. About 5–10% of these tumors give rise to malignant peripheral nerve sheath tumors (MPNSTs) which are the most common cause of mortality [10]. It is an extremely invasive sarcoma of soft tissue that metastasizes locally and grows rapidly [11]. The main treatment for MPNST is surgical excision, but this is not always possible because of the location, volume, and metastases of the tumors [12]. Only 15–66% of patients with MPNST achieve 5-year overall survival because of high insensitivity to radiation therapy and chemotherapy [13,14]. MPNSTs arise in about 8–13% of individuals with NF1 [10]. In addition to inactivation of *NF1*, the accumulation of additional genetic aberrations leads to malignant transformation into MPNST. Mutations in other genes such as epidermal growth factor receptor (*EGFR*), tumor protein p53 (*TP53*), MET proto-oncogene, receptor tyrosine kinase (*MET*) and loss of cyclin-dependent kinase inhibitor 2A (*CDKN2A*) were observed in MPNST [15].

Epidermal growth factor receptor is one of the receptor tyrosine kinase (RTK) family and is considered as a key molecule in the research of malignancy of NF1 [16]. Ligand binding to EGFR follows autophosphorylation of the receptor, which promotes a signaling cascade of downstream pathways [17]. The aberrant activation of EGFR results in cancer cell proliferation, angiogenesis, migration, and chemoresistance [18]. Overexpressed and/or hyper-activated EGFR is frequently observed in NF1-associated MPNSTs [19]. Several studies have reported that *NF1* and *TP53* mutations and MDM2 overexpression may be involved in the upregulation of EGFR expression in MPNSTs [19,20,21]. However, the precise molecular mechanisms of EGFR overexpression in NF1-associated MPNSTs are not yet understood.

In the present study, we examined the hypothesis that neurofibromin depletion may promote activation of the RAS/extracellular regulatory kinase (ERK) signaling pathway, leading to EGFR overexpression in NF1-MPNST cells. First, we assessed the expression of phosphorylated (p)EGFR in NF1-MPNST tissues. Next, we analyzed the basal expression level of EGFR in NF1-associated MPNST cells compared with that in normal cells. We found that EGFR expression was significantly increased but that of neurofibromin was significantly decreased in MPNSTs. Furthermore, our results indicate that neurofibromin deficiency causes Ras/ERK/SP1-mediated transcriptional upregulation of *EGFR* in NF1-associated MPNSTs.

## 2. Results

### 2.1. pEGFR Was Upregulated in the MPNST Tissues in Patients with NF1

Tumor tissue specimens of NF1 were obtained by surgical resection of seven patients diagnosed with NF1 at Ajou University Hospital. Clinical features of patients are shown in Table 1. Tumor stages of the tissue samples were diagnosed by IHC analysis using H&E staining, and samples from four patients (P1–P3 and P7) were diagnosed as benign PNs and three patients (P4–P6) as MPNSTs (Table 1). Since Schwann cells are considered the origin cells in both neurofibromas and MPNSTs, we performed IHC with the Schwann cell lineage marker anti-S100 antibody. The IHC images showed that all tumor specimens were positive for the S100 antibody (Table 1).

**Figure 1:**
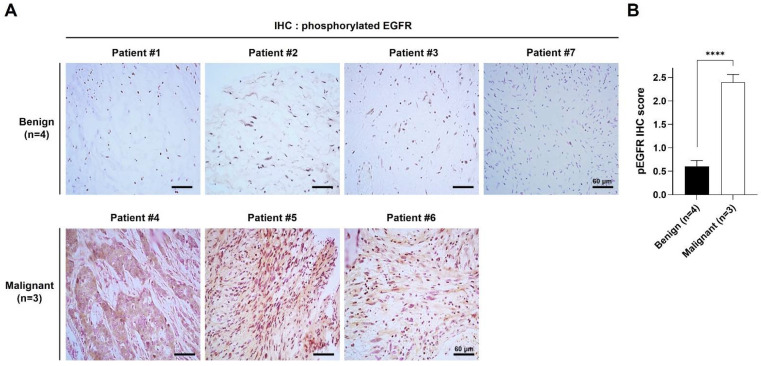
Phosphorylated EGFR is upregulated in MPNST. (**A**) Histologic analysis by immunohistochemical staining using phospho-EGFR (pEGFR) antibody was performed on tumor tissue sections. Scale bar = 60 μm (400×). (**B**) Quantitative analysis of immunohistochemical staining of pEGFR. pEGFR-positive cells (brown) and nuclei (blue) were analyzed from four different parts of each slide, and pEGFR IHC score was calculated using ImageJ software. The data are presented as mean ± standard error of mean (SEM). Two-tailed paired *t*-test was used for the statistical analysis. ****, *p* < 0.0001.

Since overexpression and hyperactivation of EGFR is often found in NF1-associated MPNST tumor tissues and cells [22], we examined the expression level of pEGFR by IHC. The positively stained level of pEGFR antibody was markedly and significantly higher in the MPNST tissues than in the PN tissues of NF1 patients (Figure 1A,B). To determine whether pEGFR and neurofibromin levels change with tumor progression, we compared their levels at two time points in the same patient. In patient P7, pEGFR and pERK1/2 levels were clearly higher in tumor tissues at age 17 years than in those at age 12 years (Table 1 and Figure S1). In addition, we observed markedly lower levels of neurofibromin in tumor tissues at age 17 years than in those at age 12 years (Figure S1).

### 2.2. EGFR Expression Level Was Inversely Corelated with Neurofibromin Expression Level in the MPNST Cells

**Figure 2:**
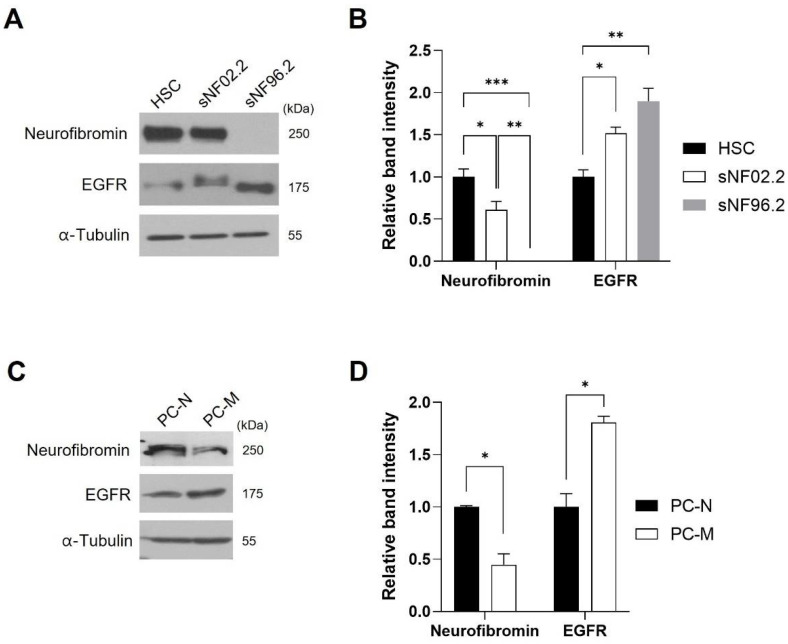
Co-expression pattern of neurofibromin and EGFR expression in MPNST cells. (**A**) Western blot analysis of neurofibromin and EGFR in HSC, sNF02.2 and NF96.2 2. (**C**) Western blot analysis of EGFR and neurofibromin in primary normal PC-N cells and malignant PC-M cells. (**B**,**D**) Immunoblot band density of specific proteins was normalized to the signal intensity of α-tubulin internal control for each sample. The western blot analysis was repeated at least three times with comparable results. Relative band intensity was quantified using ImageJ software. One-way analysis of variance (ANOVA) followed by Tukey’s honest significant difference (HSD) post-hoc test was used for the statistical analysis in (**B**), and two-tailed paired *t*-test was used for the statistical analysis in (**D**). *, *p* < 0.05, **, *p* < 0.01 and ***, *p* < 0.001.

To further investigate of expression levels of EGFR in NF1-MPNST cells, the expression levels of EGFR were examined in the three Schwann cell lines; (1) the normal human SC line (HSC) with both normal *NF1* alleles (*NF1*^+/+^), (2) sNF02.2 MPNST SC line has a missense mutation c.4868A>T (D1623V) on one mutant *NF1* allele and one normal *NF1* allele (*NF1*^+/−^), and (3) sNF96.2 2 MPNST SC line has two null alleles (a base pair deletion in exon 21 (c.3683delC), causing a frameshift mutation plus loss of heterozygosity) and no intact *NF1* allele (*NF1*^−/−^) [23,24]. The decreased expression levels of neurofibromin were found in NF1-MPNST sNF02.2 cells compared to normal HSC cells, and neurofibromin was not expressed in NF1-MPNST sNF96.2 cells (Figure 2A,B). The expression levels of EGFR were markedly upregulated in the two NF1-associated MPNST cells, compared to HSC cells (Figure 2A,B). Next, we carried out the same experiments in the NF1-associated primary cells (PC-N and PC-M) isolated from a patient with NF1. PC-N and PC-M cells carried the *NF1* nonsense mutation Y2264X (c.6792C>G) in one allele (*NF1*^+/−^), resulting in a truncated neurofibromin, as described in our previous report [25]. The basal expression levels of EGFR were higher in PC-M than PC-N, while decreased expression levels of full-length neurofibromin were observed in PC-M (Figure 2C,D).

These results indicate a strong inverse correlation between EGFR and neurofibromin expression levels in the SC lines and NF1-associated primary cultured cells.

### 2.3. Knockdown of NF1 Promotes EGFR Expression

**Figure 3:**
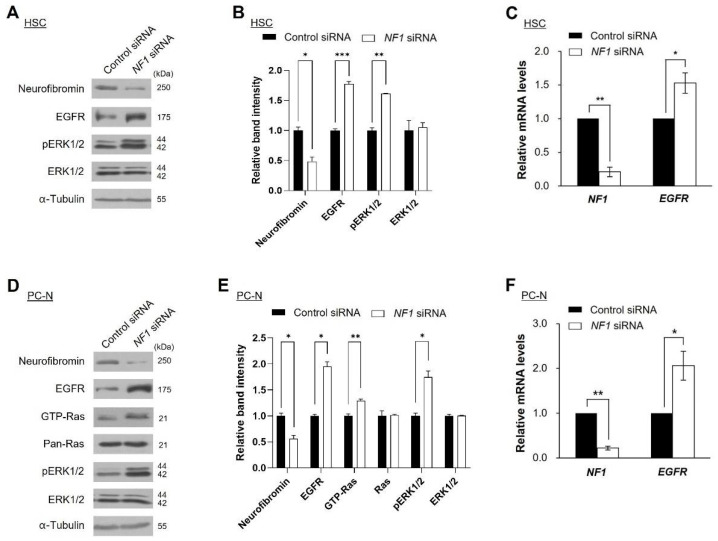
Correlation of the expression levels between neurofibromin and EGFR. The protein expressions of NF1 and EGFR were examined by western blot analyses following siRNA transfection in (**A**) human Schwann cells (HSCs) and (**D**) primary cultured normal (PC-N). (**B**,**E**) Immunoblot band density of specific proteins was normalized to the signal intensity of α-tubulin internal control for each sample. The western blot analysis was repeated at least three times with comparable results. Relative band intensity was quantified using ImageJ software. *, *p* < 0.05, **, *p* < 0.01 and ***, *p* < 0.001. (**C**,**F**) mRNA expression levels of *NF1* and *EGFR* were evaluated by real-time quantitative RT-PCR after 3 days of transfection. Two-tailed paired *t*-test was used for statistical analysis. *, *p* < 0.05 and **, *p* < 0.01.

Next, we examined whether changes in neurofibromin expression levels directly affect EGFR expression levels in NF1-associated MPNST cells. Knockdown of neurofibromin by treatment of *NF1* siRNA increased the EGFR levels in HSCs and PC-N cells (Figure 3C,F). Notably, mRNA expression levels of *EGFR* showed a close inverse correlation with the mRNA expression levels of *NF1* in HSC and PC-N cells (Figure 3C,F). These results indicate that neurofibromin influences the transcriptional regulation of *EGFR*. As neurofibromin is a negative regulator of active Ras-GTP [2], we further investigated the possible involvement of Ras/Ras-mitogen-activated protein kinase (MAPK) signaling in this correlation. Increased levels of pERK1/2 and/or activated RAS (GTP-RAS) were detected in HSCs and PC-N cells (Figure 3A,B,D,E).

### 2.4. Neurofibromin Modulates the Ras/ERK/SP1 Dependent EGFR Expression

**Figure 4:**
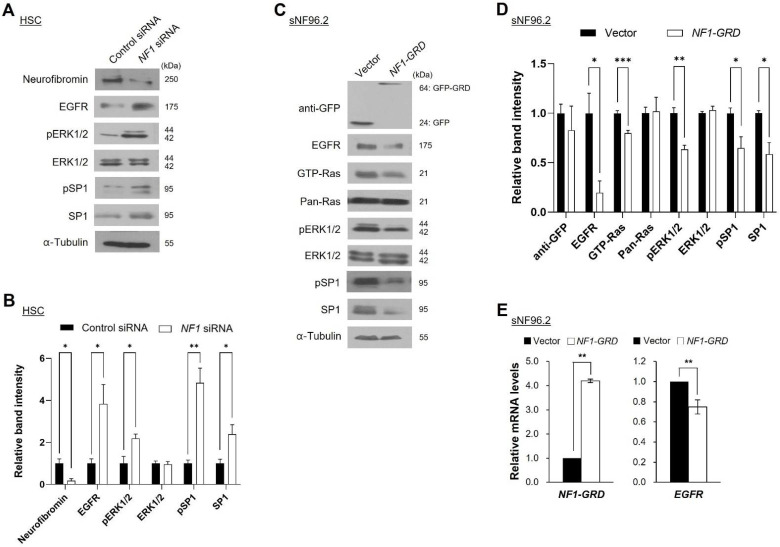
Changes in the EGFR, Ras, ERK1/2, and SP1 levels in the neurofibromin-depleted and neurofibromin-overexpressed cells. (**A**) Western blot analysis of the effect of *NF1* siRNA and its downstream signaling pathways in HSCs. (**C**) sNF96.2 cells were transfected with an empty vector or GFP-tagged *NF1-GRD*. A total of 24 h after transfection, expression levels of the indicated proteins were determined by immunoblotting. (**B**,**D**) Immunoblot band density of specific proteins was normalized to the signal intensity of α-tubulin internal control for each sample. The western blot analysis was repeated at least three times with comparable results. Relative band intensity was quantified using ImageJ software. Two-tailed paired *t*-test was used for statistical analysis. *, *p* < 0.05, **, *p* < 0.01 and ***, *p* < 0.001. (**E**) The mRNA levels of *EGFR* or *NF1-GRD* were examined by quantitative RT-PCR. Two-tailed paired *t*-test was used for statistical analysis. **, *p* < 0.01.

We further elucidated the alteration of neurofibromin-dependent EGFR expression in other cells: NF1-MPNST cells (sNF02.2 and PC-M) and normal fibroblast IMR90 cells. Depletion of neurofibromin resulted in an increase in pERK1/2 levels in all tested cells, sNF02.2, PC-M, and IMR90 (Figures S2 and S3). Next, we examined whether overexpression of neurofibromin in MPNST cells could reduce the upregulated EGFR expression. sNF96.2 cells were transfected with a GFP-tagged RAS GAP related domain of neurofibromin (NF1-GRD). *NF1-GRD* expression in the sNF96.2 cells led to inhibition of activity of RAS and pERK1/2 with a concurrent decrease of the *EGFR* mRNA levels and protein expression levels (Figure 4C–E). These results suggest that the regulation of EGFR expression is closely related to neurofibromin.

To elucidate the molecular mechanisms of neurofibromin-dependent *EGFR* transcriptional regulation, we focused on SP1, a key *EGFR* transcription factor [26]. Depletion of neurofibromin using the siRNAs increased SP1 and/or pSP1 levels in normal HSCs and IMR90 cells (Figure 4A,B and Figure S3) and sNF02.2 and PC-M MPNST cells (Figure S1). On the other hand, overexpression of *NF1-GRD* by transient transfection in sNF96.2 cells resulted in a decrease in SP1 and pSP1 levels (Figure 4C,D).

### 2.5. Inhibition of ERK1/2 Caused Downregulation of SP1 and There by Augmentation of EGFR Expression

**Figure 5:**
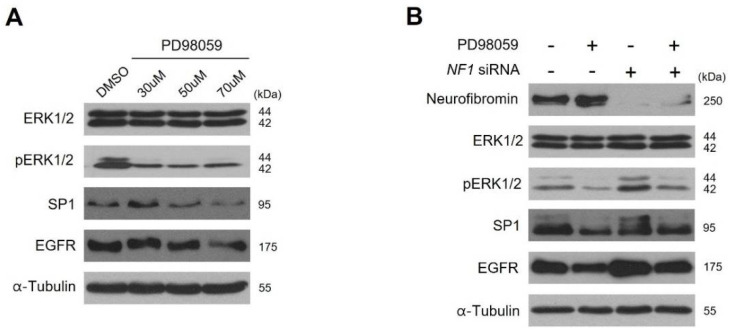
Correlation between ERK1/2 phosphorylation and SP1 and EGFR expression levels. (**A**) Human Schwan cells (HSCs) were exposed to the indicated concentrations of MAPK inhibitors PD98059 for 1 day in normal cell culture medium and protein extracts were determined by western blotting using the indicated antibodies. (**B**) HSCs were treated with *NF1* siRNA or control siRNA for 48 h followed by an additional 24 h of treatment with 70 μM PD98059. The minus (−) for PD98059 indicates DMSO treatment, and the minus (−) for *NF1* siRNA indicates control siRNA treatment. After 3 days of treatment, the levels of total α-tubulin, neurofibromin, SP1, EGFR, ERK1/2 and pERK1/2 were measured by Western blot.

Next, we examined whether SP1 plays a fundamental role in our finding of neurofibromin-dependent EGFR expression. As ERK1/2 is a major kinase for SP1 activation [27], we examined the effects of ERK1/2 inhibition on the protein levels of SP1 and EGFR in HSCs. Treatment with the ERK1/2 inhibitor PD98059 (Sigma, P215) decreased protein levels of SP1 and EGFR (Figure 5A and Figure S4A). However, in the neurofibromin-depleted HSCs, PD98059 treatment did not have an additional effect on the decreased SP1 and EGFR protein levels (Figure 5B and Figure S4B). These results directly demonstrate that neurofibromin level-dependent changes in EGFR expression are mediated by ERK1/2 and SP1 signaling.

### 2.6. Neurofibromin Regulated EGFR Expression through Controlling the Nuclear Localization and Binding Directly of SP1 to EGFR Promoter Regions

**Figure 6:**
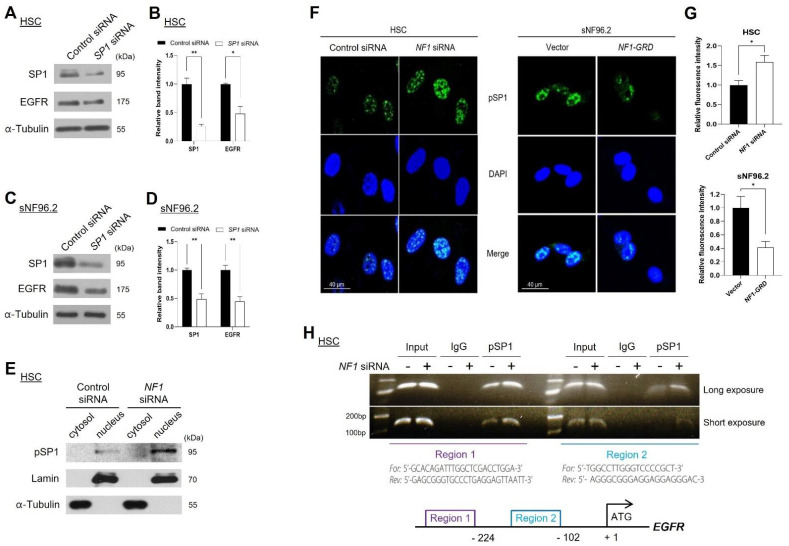
Neurofibromin-mediated regulation of a nuclear localization of phosphorylated SP1 (pSP1) and its binding to the *EGFR* gene promoter. (**A**,**C**) Western blot of SP1 and EGFR in HSC and sNF96.2 cells transfected with *SP1* siRNA or negative control siRNA. (**B**,**D**) Immunoblot band density of specific proteins was normalized to the signal intensity of α-tubulin internal control for each sample. The western blot analysis was repeated at least three times with comparable results. Relative band intensity was quantified using ImageJ software. *, *p* < 0.05 and **, *p* < 0.01. (**E**) Nuclear or cytoplasmic fractions of transfected HSCs were analyzed by immunoblotting with Lamin (nuclear marker), α-tubulin (cytoplasmic marker), and pSP1 antibodies. (**F**) HSCs were transfected with *NF1* siRNA or negative control siRNA, whereas sNF96.2 cells transfected with *NF1-GRD*. DAPI staining was performed to indicate the positions of nuclei. The cells were analyzed by confocal microscopy with an anti-pSP1 antibody. Scale bar = 40 μm. (**G**) Immunofluorescence for pSP1 (FITC, green) colocalized with DAPI (nucleus, blue) was quantified using ImageJ software. (**H**) ChIP assay was performed in HSCs transfected with *NF1* siRNA or negative control siRNA using anti-pSP1 normal rabbit IgG, and positive control. Non-immunoprecipitated chromatin (1%) was used as total input control. ChIP products were amplified by PCR using specific primers for each binding site separately. The same volume of PCR products was separated on a 2% agarose gel and the bands visualized using ethidium bromide staining.

Next, we examined the effect of expression and/or activation of SP1 on the expression of *EGFR* at the transcriptional level. Knockdown of *SP1* by siRNA treatment decreased EGFR expression in HSCs (Figure 6A,B) and sNF96.2 cells (Figure 6C,D). Activation of SP1 by phosphorylation leads to an increase in its nuclear localization, thereby pSP1 can act as a transcription factor [28]. The decrease in neurofibromin levels by *NF1* siRNA treatment resulted in an increase in pSP1 levels in the nuclear fraction of HSCs (Figure 6E) and in IMR90 cells (Figure S5A). In addition, immunocytochemistry results showed that nuclear localization of pSP1 was increased by *NF1* siRNA treatment in HSCs and IMR90 cells (Figure 6F(left),G(top) and Figure S5B), whereas it was decreased by *NF1-GRD* overexpression in sNF96.2 cells (Figure 6F(right),G(bottom)).

To determine the pSP1-binding site on the *EGFR* gene in HSCs, we conducted a site-specific ChIP assay using an anti-pSP1 antibody, as described in a previous report [29]. The ChIP assay results showed that *NF1* knockdown augmented pSP1 binding to both regions (regions 1 and 2) on the *EGFR* promoter (Figure 6H). These results indicate that neurofibromin regulates the nuclear localization of SP1 and binding of SP1 to the *EGFR* gene promoter.

### 2.7. Combined EGFR Inhibitor and Doxorubicin Treatment Showed Synergistically Enhanced Antiproliferative Effect in NF1-Deficient MPNST Cells

It is important to reduce the dosage of anticancer drugs to minimize side effects. Here, we next investigated whether the EGFR inhibitor erlotinib has a synergetic effect with the anticancer drug doxorubicin. MPNST sNF96.2 cell viability was tested under combined treatment with various concentrations of erlotinib and a low dose of doxorubicin. Notably, treatment with erlotinib along with 0.1 μg/mL of doxorubicin reduced cell proliferation in a dose-dependent manner (Figure S6).

## 3. Discussion

MPNST is the major cause of death in patients with NF1, and no adequate treatment is currently available [12]. LOH in the *NF1* gene in Schwann cells is mainly caused by large-scale somatic rearrangements, deletions, or recombination, along with germline *NF1* mutations, leading to a complete loss of neurofibromin expression, thereby resulting in tumor development [30]. Although LOH in *NF1* was observed in MPNSTs with high frequency (>four-fold) compared to benign neurofibromas, loss of NF1 is not the sole cause of the malignant progression of benign tumors to MPNSTs in patients with NF1 [31]; several studies have suggested that additional epigenetic or genetic alterations are involved in malignant transformation and tumorigenesis in NF1 [32].

Overexpression of EGFR is frequently found in NF1-associated MPNSTs [19,33] and Schwann cells [34]. Dysregulation of EGFR caused by mutation, amplification, and overexpression plays a key role in tumor progression of NF1-MPNST [18]. Increased or extended EGFR-dependent progenitor cells were found in tumor tissues of NF1-deficient mice [35] and upregulation of EGFR stimulates the activation of the downstream signaling, the MAPK cascade [36]. Collectively, the upregulation of EGFR expression is a key factor for malignant progression from benign PNs to MPNSTs in NF1. However, the molecular mechanisms of EGFR dysregulation in NF1-associated MPNSTs remain to be elucidated.

Our results indicate that the decrease in neurofibromin is directly related to the level of EGFR expression. First, although there is a limitation in patient samples, we found that reduced neurofibromin in patient-derived NF1-associated MPNST tissues and MPNST cells are the main cause of the overexpression of EGFR. Collectively, the upregulation of EGFR expression is a key factor for malignant progression from benign PNs to MPNSTs in NF1. However, the molecular mechanisms of EGFR dysregulation in NF1-associated MPNSTs remain to be elucidated. Primary cultured cells from a patient with NF1 and Schwann cell lines revealed a lower level of neurofibromin and higher level of EGFR in the MPNSTs (Figure 2). Second, manipulation of neurofibromin expression by *NF1* siRNA treatment in normal and malignant MPNST cells and *NF1-GRD* overexpression in malignant MPNST cells showed a perfect inverse correlation between neurofibromin and EGFR levels at both the mRNA and protein levels (Figure 2, Figure 3 and Figure 4). These results suggest that overexpression of EGFR found in MPNSTs may be caused by an insufficient role of neurofibromin due to mutation, LOH, or reduction of expression in the *NF1* gene.

Importantly, this work discovered the novel molecular mechanism of the neurofibromin-mediated regulation of EGFR. Insufficiency or loss of neurofibromin in the NF1-associated MPNSTs causes hyperactivation of the Ras/ERK signaling pathway, followed by enhancement of the activated (phosphorylated) SP1 translocation from the cytosol to the nucleus and binding to the *EGFR* promoter regions, resulting in the augmentation of *EGFR* transcription. SP1 is one of the main transcription factors for the *EGFR* gene. Phosphorylation of SP1 by pERK1/2 enhances the transcriptional activity of SP1, which further enhances the binding of pSP1 to the *EGFR* promoter [27,37]. In addition, our results showed that ERK1/2 is responsible for the phosphorylation of SP1 (Figure 5). Notably, our ChIP assay results showed that SP1-mediated regulation of *EGFR* transcription was closely dependent on neurofibromin levels (Figure 6). Thus, ERK inhibition may suppress the nuclear localization of SP1 and in turn, the binding of pSP1 to the *EGFR* promoter. These results can explain how neurofibromin regulates EGFR expression; reduction of neurofibromin levels induces the activation of the Ras/ERK signaling pathway, leading to an increase in SP1 phosphorylation and binding to the *EGFR* gene promoter region and subsequently promoting *EGFR* transcription.

Downregulation of neurofibromin expression levels has been suggested to be involved in the resistance to anticancer drugs caused by hyperactivation of Ras/ERK signaling in NF1-associated MPNST cells [38], clear cell kidney cancer cells [39], mouse embryonic fibroblasts, and mouse neurofibroma-associated Schwann cells [40]. EGFR dysregulation contributes to MPNST transformation by promoting the proliferation of Schwann cells in NF1 and induces drug resistance in MPNST [41]. Furthermore, overexpression of EGFR is strongly linked with poor prognosis and overall survival of patients with MPNST [42]. The EGFR/Ras and mTOR signaling pathways are major targets for the development of chemotherapeutic drugs for MPNSTs [43]. The EGFR inhibitor erlotinib, which binds to the kinase domain of EGFR, has already been implicated in NF1-associated MPNST [44]. However, the EGFR inhibitor erlotinib alone was ineffective in clinical trials of MPNST [45]. Chemotherapy based on the anticancer drug doxorubicin is the standard medication for the treatment of MPNST [46], although side effects have been reported [47]. Doxorubicin is widely used for the treatment of several types of cancers; however, a high risk of cardiac toxicity has been reported with the use of high doses [48]. Therefore, it is important to reduce the dosage of doxorubicin to minimize side effects. We tested a combination of erlotinib and doxorubicin in malignant MPNST cells. Under a low dose of doxorubicin (0.1 μg/mL), combined treatment with erlotinib showed a synergistic effect on the proliferation of MPNST cells in a dose-dependent manner (Figure S6).

Reduced neurofibromin and activated and overexpressed EGFR were markedly observed in NF1-associated MPNST cells. This is the first study to demonstrate the molecular mechanism of neurofibromin-deficiency-mediated EGFR upregulation in NF1-associated MPNSTs. In summary, our results indicate that deficiency of neurofibromin causes Ras/ERK/SP1-mediated transcriptional upregulation of EGFR in NF1-associated MPNST. Our results may provide a basis for the progression of benign tumors and novel therapeutic strategies for NF1-associated MPNSTs.

## 4. Materials and Methods

### 4.1. Cell Lines

Primary malignant MPNST cells (PC-M) and normal phenotypic cells (PC-N) isolated from a patient with NF1 were cultured as previously described [25]. The human normal fibroblast IMR90, sNF02.2 MPNST cell lines and sNF96.2 MPNST cell lines were obtained from American Type Culture Collection and incubated in DMEM (HyClone, Logan, UT, USA, SH30022.01) with 100 U/mL penicillin, 10% fetal bovine serum (Gibco, Waltham, MA, USA, 16000-044) and 100 μg/mL streptomycin. Human normal Schwann cells (HSCs) were purchased from ScienCell Research Laboratories and maintained in Schwann cell medium (#1701, Sciencell) supplemented with 1% Schwann cell growth supplement (#1752, ScienCell), 100 U/mL penicillin, 10% fetal bovine serum, and 100 μg/mL streptomycin.

### 4.2. Patient Samples

This work was approved by the Institutional Review Board of Ajou University Hospital (AJIRB-GEN-GEN-11-321 and AJIRB-GEN-SMP-11-107) and following informed patient consent. Seven NF1-related tumor tissue samples consisting of 4 PNs, and 3 MPNSTs samples from patients diagnosed with NF1 were obtained from the Ajou University Hospital Pathology Department.

### 4.3. Cell Proliferation Assays

A total of 4 × 10^3^ cells were cultured into 96-well plates per well 1 day before drug treatment. After 24 h of drug treatment, 10 µL EZ-Cytox (EZ-1000, DoGenBio, Seoul, Korea) was added, and the plates were incubated at 37 °C for 2 h. Absorbance was recorded with a microplate reader at a wavelength of 450 nm (Model 680 microplate reader, Bio-Rad Laboratories Inc., Hercules, CA, USA).

### 4.4. Subcellular Fractionations and Western Blot

Cells were harvested with hypotonic buffer (25 mM NaCl, 20 mM HEPES, 20% glycerol, pH 7.4, 10 mM KCl, 1 mM EDTA, 1.5 mM MgCl_2_, 1 mM DTT and 0.1% Triton X100), incubated at 4 °C for 20 min and homogenized with a Dunce homogenizer for 20 strokes. The homogenate was centrifuged for 10 min at 3000 rpm and the supernatant was obtained as the cytoplasmic fraction. The nuclear fraction was lysed in lysis buffer (pH 7.4, 20 mM HEPES, 300 mM NaCl, 10 mM KCl, 1.5 mM MgCl_2_, 1 mM EDTA, 20% glycerol, 1 mM DTT and 0.1% Triton X100), at 4 °C for 30 m and centrifuged at 13,200 rpm for 10 min. The supernatant containing the nuclear fraction was collected.

Total proteins were isolated using RIPA buffer with 1 mM phenylmethylsulfonyl fluoride (Roche, 10837091001) and a protease inhibitor cocktail (Roche Applied Science, Penzberg, Germany, 11836170001). The concentration of extracted protein was quantified using the Bradford assay (Bio-Rad Protein Assays Kits, #5000001). The prepared protein samples (20–40 μg) were loaded on 8–15% sodium dodecyl sulfate-polyacrylamide gel electrophoresis (SDS-PAGE) and were filtered through a PVDF membrane (Millipore, Billerica, MA, USA) followed by 1 h of blocking in 5% skim milk with Tween-20. The PVDF membranes were incubated overnight with primary antibodies against EGFR (Cell Signaling Technology, Danvers, MA, USA, #2232), P-EGFR (Epitomics Inc., Burlingame, CA, USA, 1727-1), ERK1/2 (Cell Signaling Technology, Danvers, MA, USA,, #9102), P-ERK1/2 (Cell Signaling Technology, Massachusetts, MA, USA,, #4376), Neurofibromin (Santa Cruz Biotechnology, Santa Cruz, CA, USA, sc-67), α-tubulin (Santa Cruz Biotechnology, Santa Cruz, CA, USA, sc-5286), SP1 (Santa Cruz Biotechnology, Santa Cruz, CA, USA, sc-17824), phosphorylated SP1 (pSP1) (Abcam, Cambridge, MA, USA, ab5925), GFP (Thermo Fisher Scientific, MA, USA, #MA5-15256), lamin B (Sigma, St Louis, MO, USA, SAB1306342), and S100 (Thermo Fisher Scientific, MA, USA, #MA5-12969). Following reaction with secondary anti-rabbit (Santa Cruz, sc-2004) or anti-mouse antibodies (Santa Cruz Biotechnology, Santa Cruz, CA, USA, sc-2005), immunoreactive bands were imaged using enhanced chemiluminescence (ECL) blotting detection reagents (Intron Biotechnology, Seongnam-si, Gyeonggi-do, Korea, 16028). Relative band intensity of specific proteins was quantified after normalization using ImageJ software (National Institutes of Health, Bethesda, MD, USA).

### 4.5. Chromatin Immunoprecipitation (ChIP) Assay

ChIP and PCR were performed as described earlier [29]. Cells were treated in 1% formaldehyde for 10 min at RT to crosslink between proteins and DNA and then quenched by adding 1.25 M glycine. Cells were washed with 1× PBS, collected, and sheared using a Vibracell sonicator (SONICS & Materials, Danbury, CT, USA) to a size of 100–1000 bp. Lysates were precleared with A/G PLUS-Agaros and incubated at 4 °C overnight with 2 μg of normal rabbit immunoglobulins or pSP1 antibody. Non-immunoprecipitated chromatin was used as total input control. The input chromatin or chromatin immunoprecipitated using an antibody (2 μg of SP1 or IgG) was subjected to PCR analysis. The DNA–protein–antibody complexes were then washed with RIPA buffer and elution buffer was used for DNA elution (100 mM NaHCO_3_, 1% SDS). After cross-linking reversal at 65 °C for 4 h, DNA was isolated using DNA purification kit from Qiagen (QIAGEN, Hilden, Germany, 28106) and used as a template for PCR of the *EGFR* promoter. The primers were A: 5′-GCACAGATTTGGCTCGACCTGGA-3′ and 5′-GAGCGG GTGCCCTGAGGAGTTAATT-3′; B: 5′-TGGCCTTGGGTCCCCGCT-3′ and 5′-AGGGCG GGAGGAGGAGGGAC-3′.

### 4.6. Immunocytochemistry

The cells were plated in chambers and cultured at 37 °C in a 5% CO_2_ incubator for 24 h. Then, cells were rinsed with PBS and fixed at RT in 4% paraformaldehyde for 10 min. For cell fixation, cells were rinsed three times in 1× PBS and permeabilized in PBS with 0.5% Triton X-100 for 10 min followed by blocking with 1% BSA for 1 h. The cells were incubated at RT with primary antibody for 1 h. After being washed twice with 1× PBS, fixed cells were reacted with FITC-labeled secondary antibody. Samples were mounted using mounting solution including DAPI (Vector Laboratories, Burlingame, CA, USA, H-1200) and imaged under a fluorescence confocal microscope (LSM 710, Carl Zeiss). Immunofluorescence for pSP1 (FITC, green) colocalized with DAPI (nucleus, blue) was quantified using ImageJ software (National Institutes of Health, Bethesda, MD, USA).

### 4.7. Immunohistochemistry (IHC)

Tumor tissue samples obtained from patients with NF1 were fixed with 10% formalin and were embedded in paraffin blocks. Then, 10 µm consecutive sections were prepared, deparaffinized and rehydrated. Antigen retrieval was performed by heating slides with 10 mM sodium citrate buffer. Then, the sections were incubated with 3% hydrogen for 5 min to inactivate endogenous peroxidases. Tissues were blocked using Ultra V-Block (Thermo Scientific) and incubated at RT with primary antibodies for 1 h. The slides were treated with HRP polymer conjugated secondary antibody and visualized by 3,3-diaminobenzidine. Nuclear counterstaining was carried out with Mayer’s hematoxylin. To quantify the immunoreactivity, IHC slides were divided into four different areas according to the staining intensity. The pEGFR IHC score was calculated as pEGFR (brown; diaminobenzidine)-positive cells divided by nucleus (blue)-positive total cells counted using ImageJ software (National Institutes of Health, Bethesda, MD, USA).

### 4.8. Plasmids, Short Interfering RNAs (siRNAs), and Transfection

*SP1* siRNA targeting sequences, 5′-GCAACATGGGAATTATGAA-3′ and *NF1* siRNA targeting sequences, 5′-CAGTGAACGTAAGGGTTCT-3′ were synthesized by Genolution Pharmaceuticals. Scrambled RNA was obtained from Santa Cruz Biotechnology. For siRNA transfection, cells were transfected with targeted siRNA and negative control siRNA using Lipofectamine RNAiMax reagent (Invitrogen, Shanghai, China, 13778-075). The GFP-tagged *NF1-GRD* plasmid was used as described previously [49]. The plasmids were transfected into cells using the Lipofectamine2000 transfection agent (Invitrogen, 11668-019), according to the manufacturer’s instructions.

### 4.9. Quantitative Real-Time Reverse Transcription-Polymerase Chain Reaction (qRT-PCR)

Total RNA from cells was extracted using TRIzol reagent (Invitrogen, 15596026) and quantified using Nanodrop. cDNAs were synthesized using the Revert Aid H Minus First Strand cDNA Synthesis kit (Fermentas, Burlington, ON, Canada, K1632). Subsequent PCRs were carried out using the SYBR Premix Ex Taq (Takara Co., Otsu, Japan, #RR420A) on an ABI 7500 Fast Real-Time PCR System. The PCR conditions were as follows: firstly, 95 °C for 5 min; secondly, 95 °C for 5 s and 58 °C for 25 s; and finally, 72 °C for 30 s. In total there were 40 thermal cycles. *GAPDH* mRNA level was used as an internal reference for data normalization. Primers used were the p187403 primer set (Bioneer, Daedeok-gu, Daejeon, Korea) for the *EGFR*, P238284 primer set (Bioneer, Korea) for *NF1*, 5′-GCGATGGCTCTGGCCAATGTG-3′ and 5′-GAGAGTCTGCATGGAGTCTGCCA-3 for *NF1-GRD*, and 5′-TGTTGCCATCAATGACCCCTT-3′ and 5′-CTCCAC GACGTACTCAGCG-3′ for the *GAPDH* gene.

### 4.10. Ras Activation Assay

Ras activity was assessed using the Ras Activation Assay Kit (Upstate Biotechnology, Lake Placid, NY, USA, #17–218), according to the manufacturer’s protocol. Briefly, cells were lysed with lysis buffer. A total of 300 μg of protein was isolated for immunoprecipitation of the activated form of Ras (GTP-Ras) with Raf-1-RBD agarose beads at 4 °C. The beads were rinsed three times with supplied washing buffer and then eluted by boiling with a Laemmli sample buffer. Proteins were subjected to SDS–PAGE and immunoblotted.

### 4.11. Statistical Analysis

The bar graphs illustrate the mean ± standard error of the mean (SEM). Statistical analyses were performed using SPSS 11.0 for Windows (SPSS, Inc., Chicago, IL, USA). Statistically significant differences between groups were analyzed using the Student’s *t*-test. Comparisons of multiple groups were performed using one-way analysis of variance (ANOVA) followed by Tukey’s honest significant difference (HSD) post-hoc test. *p* < 0.05 was considered to indicate statistical significance.

## 5. Conclusions

We found that reduced neurofibromin in NF1-associated MPNST cells was the main cause of the overexpression of EGFR. Comparison of primary tissue-cultured normal cells and malignant cells from the same patient with NF1 showed a perfect inverse correlation between neurofibromin and EGFR expression levels, strongly suggesting that neurofibromin-mediated hyperactivation of EGFR is responsible for tumor progression of PNs to MPNSTs. Insufficiency or loss of neurofibromin in the NF1-associated MPNSTs causes hyperactivation of the Ras/ERK/SP1 signaling pathway, followed by enhancement of the activated (phosphorylated) SP1 translocation from the cytosol to the nucleus and binding to the *EGFR* promoter regions, resulting in the augmentation of *EGFR* transcription.

## Figures and Tables

**Table 1:** Histological findings and clinical characteristics of seven patients with neurofibromatosis type 1, including tumor tissue samples from patient #7 at two different time points (at ages 12 and 17 years).

Patients			Histological Findings			Clinical Features							Genotype
ID	Gender	Age at Diagnosis	H&E	S100	pEGFR	Café-au-Lait Spots	Neuro-Fibromas	Freckling	Optic Glioma	Lisch Nodule	Skeletal Dysplasia	Family History	*NF1* Mutation
P1	Male	59	Benign	+	+	Y	Y	Y	N	N	N	Y	N/A
P2	Male	42	Benign	+	+	Y	Y	Y	N	N	N	Y	N/A
P3	Female	5	Benign	+	+	Y	Y	Y	N	N	N	Y	N/A
P4	Male	39	Malignant	+	++	Y	Y	N	N	N	N	N	N/A
P5	Male	32	Malignant	+	++	Y	Y	N	N	N	N	N	c.4861_4862 GT>AG
P6	Female	41	Malignant	+	++	Y	Y	Y	N	N	N	N	N/A
P7	Male	12	Benign	+	+	Y	Y	Y	N	Y	Y	N	c.4537C>T
		17	Benign	+	++	Y	Y	Y	N	Y	Y	N	c.4537C>T

Abbreviations: NF1, neurofibromatosis type1; H&E, hematoxylin and eosin; pEGFR, phosphorylated EGFR; N/A, not analyzed; +, lower expression; ++, higher expression; Y, the patient had the indicated clinical features; N, the patient had not the indicated clinical features

## Data Availability

The data presented in this study are available on request from the corresponding author.

## Supplementary Materials

The Supplementary Materials are available online: https://www.mdpi.com/article/10.3390/ijms222413308/s1.
