# Supplementary material for: Neurofibromin Deficiency Causes Epidermal Growth Factor Receptor Upregulation through the Activation of Ras/ERK/SP1 Signaling Pathway in Neurofibromatosis Type 1-Associated Malignant Peripheral Nerve Sheet Tumor
Source: Int J Mol Sci. 2021 Dec 10;22(24):13308. doi: 10.3390/ijms222413308 (PMC8706697; doi:10.3390/ijms222413308)
Supplement: Supplementary file 1 [file ijms-22-13308-s001.zip › ijms-1504169-supplementary.pdf]

Neurofibromin Deficiency Causes Epidermal Growth Factor Receptor Upregulation through the Activation of Ras/ERK/SP1 Signaling Pathway in Neurofibromatosis Type 1-Associated Malignant Peripheral Nerve Sheet Tumor

Gun-Hoo Park [1,2,†], Su-Jin Lee [1,2,†], Chang-Gun Lee [1,2], Jeonghyun Kim [1,2], Eunkuk Park [1,2,*] and Seon-Yong Jeong [1,2,*]

[1] Department of Medical Genetics, Ajou University School of Medicine, Suwon 16499, Korea; gunhoopark5699@aumc.ac.kr (G.-H.P.); sujinlee5699@gmail.com (S.-J.L.); dangsunsang@ajou.ac.kr (C.-G.L.); kjh2828@ajou.ac.kr (J.K.)

[2] Department of Biomedical Sciences, Ajou University Graduate School of Medicine, Suwon 16499, Korea

* Correspondence: jude0815@ajou.ac.kr (E.P.); jeongsy@ajou.ac.kr (S.-Y.J.); Tel.: +82-31-219-4520 (E.P. & S.-Y.J.)

† These authors contributed equally to this work.

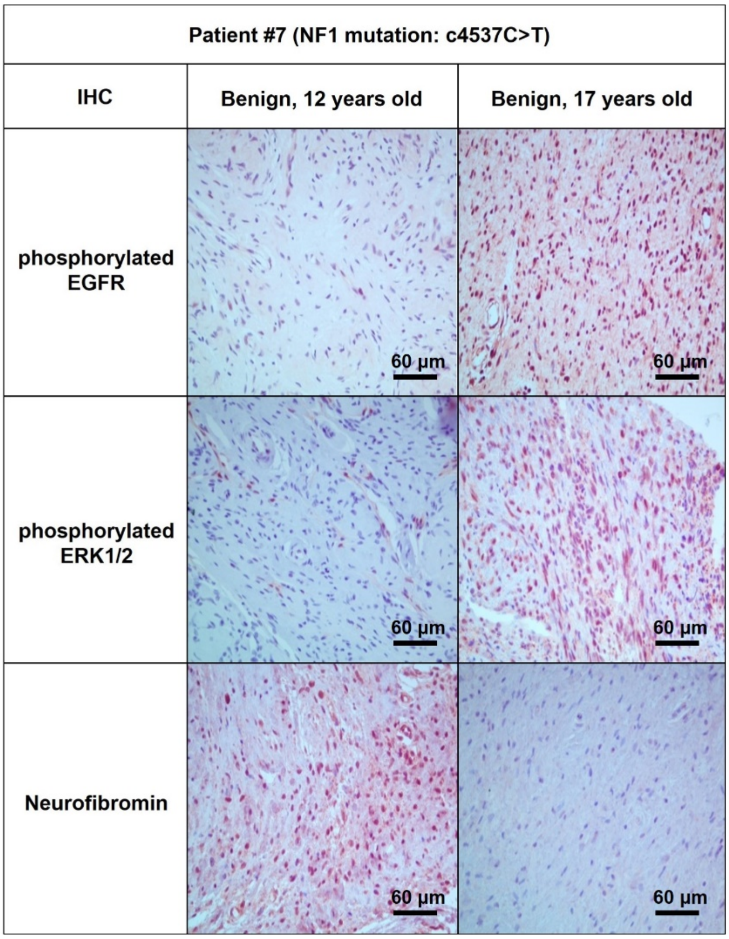

Supplementary Figure S1. Immunohistochemical staining (IHC) in the tumor tissues from a patient with NF1. Histologic analysis by IHC using antibodies against phospho-EGFR, phospho-ERK1/2 and neurofibromin were carried out at two different time points PN tissue sections. Scale bar = 60 µm (400x).

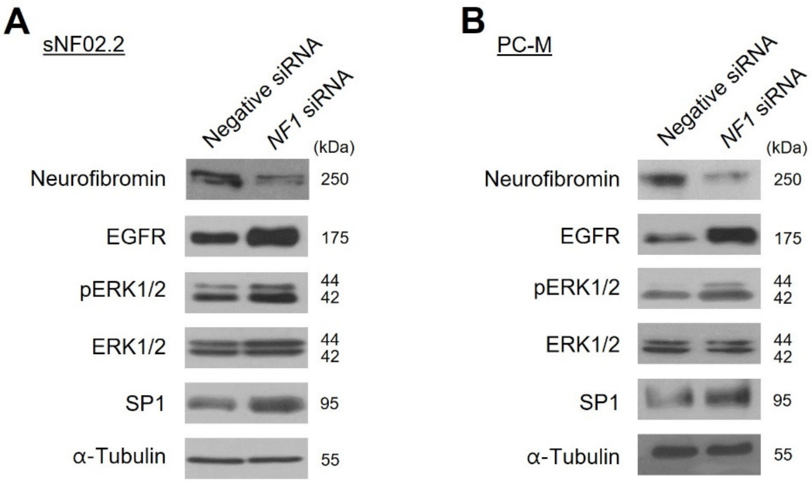

Supplementary Figure S2. Increase in expression levels of EGFR, pERK1/2, and SP1 in the *NF1*-depleted malignant peripheral nerve sheet tumor cells. (**A**) sNF02.2 cells and (**B**) PC-M cells were transfected with siRNAs (100 nM) against *NF1* gene or the nonspecific negative control. After 72 h incubation, protein levels of the indicated proteins were determined by western blotting analysis. *α*-tubulin protein level was used as the internal control.

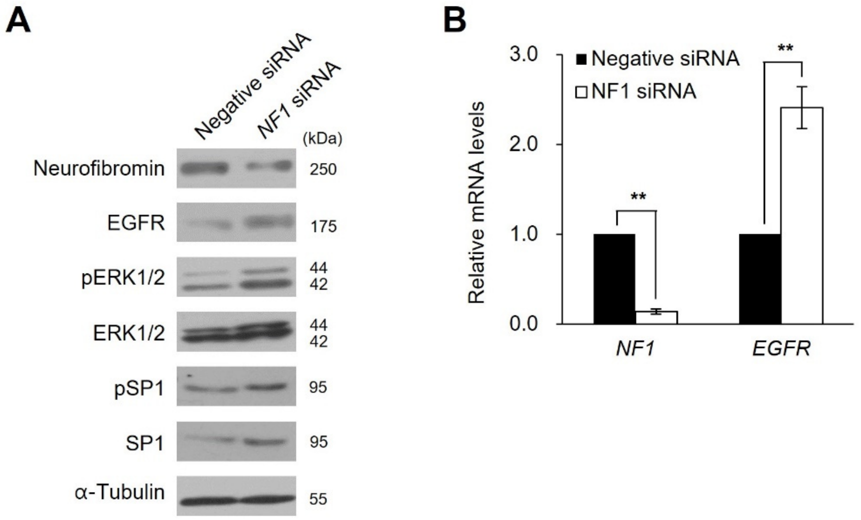

Supplementary Figure S3. Increase in the expression levels of EGFR, pERK1/2, SP1, and pSP1 in the *NF1*-depleted normal IMR90 cells. (**A**) IMR90 cells were transfected with siRNAs gene (100 nM) against *NF1* or the nonspecific negative control. After 72 h incubation, protein levels of the indicated proteins were determined by western blotting analysis. α-tubulin protein level was used as the internal control. (**B**) Relative *NF1* and *EGFR* mRNA levels in IMR90 cells were assessed by real-time reverse transcription polymerase chain reaction. Two-tailed paired t-test was used for the statistical analysis. *, $p < 0.05$ and **, $p < 0.01$.

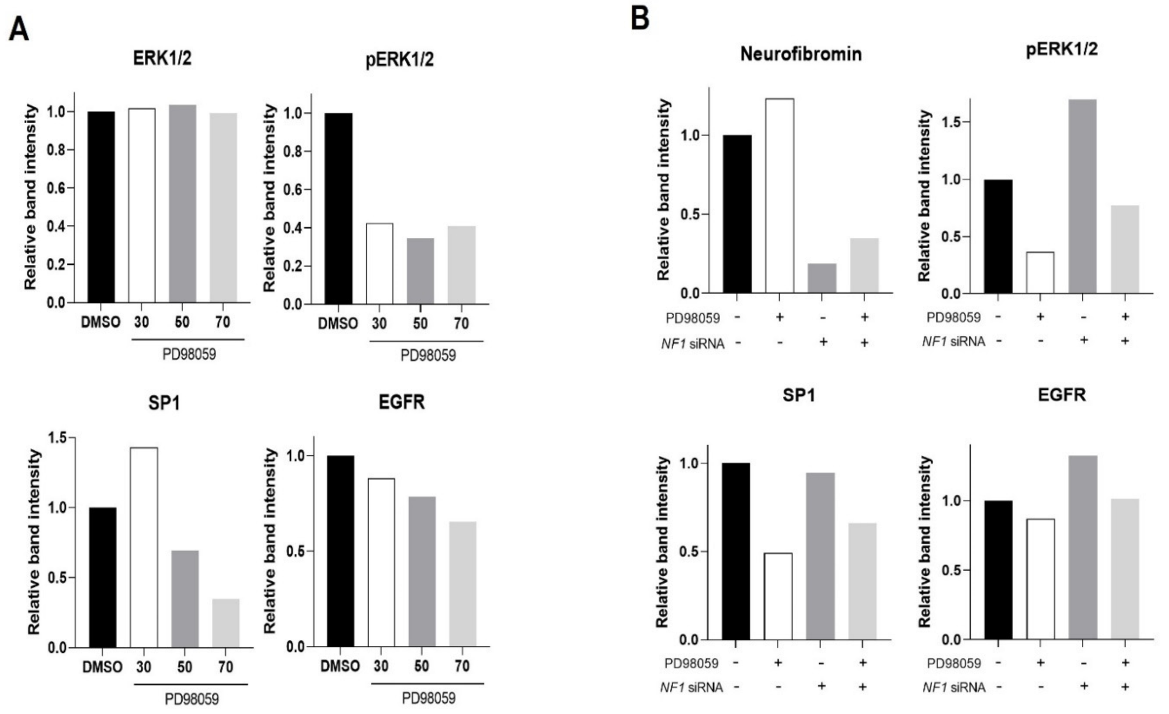

Supplementary Figure S4. Relative band intensity of correlation between ERK1/2 phosphorylation and SP1 and EGFR expression levels. (A) HSCs were exposed to the indicated concentrations of MAPK inhibitors PD98059 for 1 day in normal cell culture medium and protein extracts were determined by western blotting using the indicated antibodies. (B) HSCs were treated with *NF1* siRNA in combination with 70 μM of PD98059. The minus for PD98059 indicated DMSO and the minus for *NF1* siRNA indicated negative siRNA treatment After 3 days of treatment, the levels of total α-tubulin, neurofibromin, SP1, EGFR, ERK1/2 and pERK1/2 were measured by Western blot. Immunoblot band density was normalized by the signal intensity of α-tubulin internal control for each sample using ImageJ software.

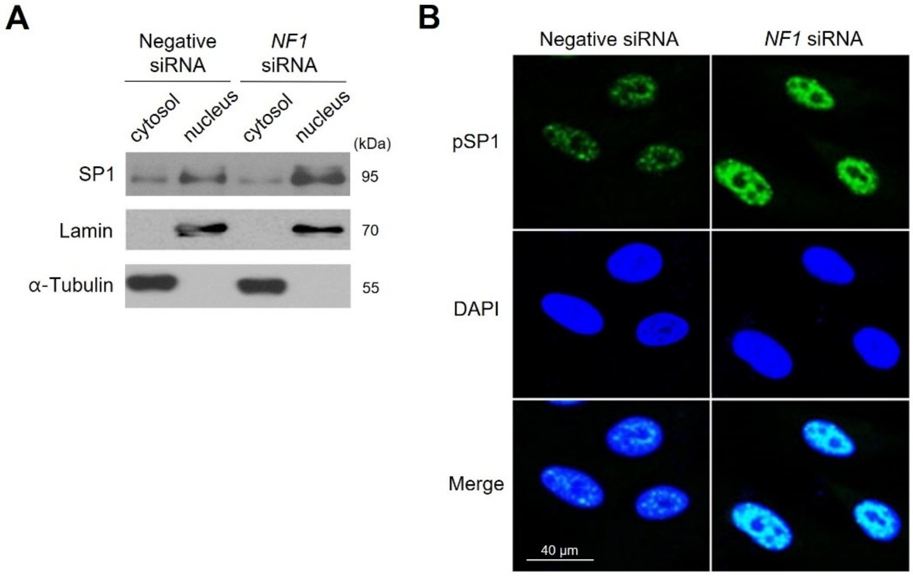

Supplementary Figure S5. Increase in nuclear localization of phosphorylated SP1 (pSP1) in the neurofibromin-depleted normal IMR90 cells. (**A**) Cellular fraction of SP1 in the neurofibromin-depleted IMR90 cells. Cells were transfected with siRNAs against the *NF1* gene (100 nM) or the nonspecific negative control (100 nM). After 72 h incubation, cells were harvested and homogenized. The whole cell extract was divided into the cytoplasmic and nuclear fractions. The SP1, lamin (nuclear marker), and α-tubulin (cytosol marker) were assessed by western blotting analysis. (**B**) Confocal microscope images of nuclear localizing pSP1. IMR90 cells were transfected with siRNAs against the *NF1* gene (100 nM) or the nonspecific negative control (100 nM) and then incubated for 72 h. Cells were further treated with anti-pSP1 antibody and mounting media containing DAPI. Scale bar = 40 μm. Immunocytochemistry images were captured and analyzed using LSM 710 confocal microscope.

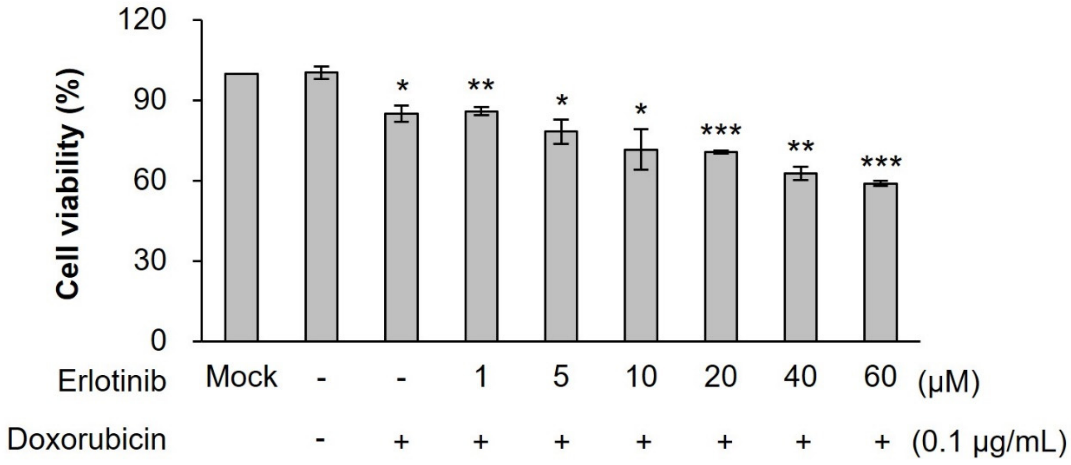

Supplementary Figure S6. Antiproliferative effect of the combined treatment of EGFR inhibitor erlotinib and doxorubicin in the NF1-depleted malignant peripheral nerve sheet tumor cells. sNF96.2 cells were treated with doxorubicin (Sigma, D1515) alone or co-treated with the indicated concentrations of erlotinib (Selleck chemicals, S7786) for 24 h. Untreated cells (Mock) were used as controls. The minus indicated DMSO solvent. Cell viability was assessed by the Ez-Cytox assay. One-way analysis of variance (ANOVA), followed by Tukey's honest significant difference (HSD) post-hoc test was used for the statistical analysis. *$p < 0.05$, **$p < 0.01$, and ***$p < 0.001$ vs. Mock.
